# Task, muscle and frequency dependent vestibular control of posture

**DOI:** 10.3389/fnint.2014.00094

**Published:** 2015-01-09

**Authors:** Patrick A. Forbes, Gunter P. Siegmund, Alfred C. Schouten, Jean-Sébastien Blouin

**Affiliations:** ^1^Department of Biomechanical Engineering, Faculty of Mechanical, Maritime and Materials Engineering, Delft University of TechnologyDelft, Netherlands; ^2^School of Kinesiology, University of British ColumbiaVancouver, B. C., Canada; ^3^MEA Forensic Engineers & ScientistsRichmond, B. C., Canada; ^4^Laboratory of Biomechanical Engineering, Institute for Biomedical Technology and Technical Medicine (MIRA), University of TwenteTwente, Netherlands; ^5^Institute for Computing, Information and Cognitive Systems (ICICS), University of British ColumbiaVancouver, B. C., Canada; ^6^Brain Research Centre, University of British ColumbiaVancouver, B. C., Canada

**Keywords:** vestibular reflexes, postural control, task dependent, frequency response, appendicular muscles, axial muscles

## Abstract

The vestibular system is crucial for postural control; however there are considerable differences in the task dependence and frequency response of vestibular reflexes in appendicular and axial muscles. For example, vestibular reflexes are only evoked in appendicular muscles when vestibular information is relevant to postural control, while in neck muscles they are maintained regardless of the requirement to maintain head on trunk balance. Recent investigations have also shown that the bandwidth of vestibular input on neck muscles is much broader than appendicular muscles (up to a factor of 3). This result challenges the notion that vestibular reflexes only contribute to postural control across the behavioral and physiological frequency range of the vestibular organ (i.e., 0–20 Hz). In this review, we explore and integrate these task-, muscle- and frequency-related differences in the vestibular system’s contribution to posture, and propose that the human nervous system has adapted vestibular signals to match the mechanical properties of the system that each group of muscles controls.

## Introduction

The vestibular system senses linear and angular head motion in space. This sensory information is used by the central nervous system to elicit reflexes and control appendicular, axial and extraocular muscles that are crucial for posture and gaze. Vestibular reflexes vary across and within muscle groups and are modulated by spatial and temporal factors related to a muscle’s contribution to the system dynamics, the different neural pathways innervating each muscle, and the congruency of sensory signals and motor commands for a given task. Recent findings from our lab indicate that these modulating mechanisms may be related to the frequency content of the vestibular signals impinging on the different muscles. Like many electromechanical systems, the vestibular system’s input-output response varies with stimulus frequency, and like many biological systems, the bandwidth of this frequency response has evolved to match the mechanical system being controlled. This review examines the frequency response of the vestibular system’s reflexive control of posture. More specifically, it focuses on the differences in the frequency response and task dependence of vestibular reflexes controlling appendicular and spinal muscles in order to better understand the neurophysiological principles governing how humans achieve stable upright posture of the head and body. We argue that the frequency response of vestibular reflexes is governed by the mechanical systems under their control, with the neck system exhibiting a broader bandwidth than the appendicular muscles. The higher frequency response in neck muscles can be modulated but not inhibited, and in contrast to the lower frequency response observed in the appendicular muscles, its contribution to muscle activity does not dependent on a neck muscle’s contribution to postural control. Based on this evidence, we propose the higher frequency response of the vestibulocollic reflexes (VCR) is functionally similar to the vestibulo-ocular response for the coordination of eye-head movements as well as head postural control during gaze shifts.

## Frequency response of the vestibular system

**Figure 1:**
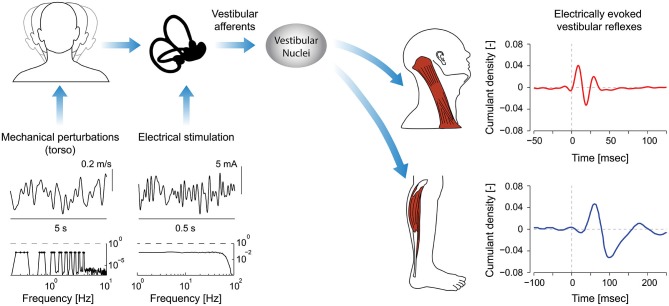
**Signal processing pathways and evoked reflex responses as a result of mechanical perturbations and/or electrical stimulation**. Input stimuli span specific bandwidths (see spectra, mechanical: 0–4 Hz; electrical stimulation: 0–75 Hz) and when applied to the head generate vestibular afferent activity. Note: the representative mechanical perturbation is applied via the torso and limited to less than 10 Hz on account of the bandwidth of mechanical device applying the perturbation and the biomechanics of the human body. Consequently, the evoked responses in axial and appendicular muscles are presented only in response to electrical stimuli with identical bandwidths (0–75 Hz). Afferent signals descend through the vestibular nuclei (VN) to axial and appendicular muscle motoneurons. The evoked reflexes in axial muscles have much shorter latencies than those in appendicular muscles (8 ms vs. 50 ms). Different time scales were used to illustrate the evoked responses (250 ms vs. 150 ms). (Data for cumulant density plots and perturbation/stimulation signals are adapted from Forbes et al., 2013a,b, 2014).

The frequency response of the vestibular system is governed by the electromechanical properties of the sensory organs and the various neural structures and pathways that then carry these sensory signals to motor neurons (see Figure 1). As described in more detail below, these properties appear to be tuned to generate response characteristics specific to the biomechanical system being controlled by those muscles.

Vestibular signals originate from two types of sensory organs: the otolith organs, which encode linear motion, and the semi-circular canals, which encode angular motion. Two otolith organs and three semi-circular canals are contained in each of the two vestibular apparatus. Otolith afferents demonstrate dynamic responses that are in phase with linear acceleration, whereas semi-circular canal afferents demonstrate dynamic responses that are in phase with angular velocity at frequencies above about 0.1 Hz and up to 4.0 Hz (Goldberg et al., 2012).

Animal and human studies have reported that normal head motion, and thus the stimulus for the vestibular system, has relatively low frequency content. Animals performing voluntary head movements (with or without gaze redirections) exhibit head rotational velocity profiles containing frequency information approaching 20 Hz (Armand and Minor, 2001; Huterer and Cullen, 2002). Similarly, head rotational velocity in humans performing locomotor tasks exhibit frequency content up to 20 Hz (Grossman et al., 1988; Pozzo et al., 1990). Highly active movements, such as running, playing sports, and jumping, seem to increase this bandwidth to 30 Hz or higher (Carriot et al., 2014).

While the measured frequency bandwidth of head motion has been limited to 0–30 Hz, the vestibular afferents are capable of much higher frequency response dynamics. The frequency response (i.e., gain and phase) of turtle otoliths during inertial stimuli reaches 500 Hz and resembles that of a linear second order system with a natural frequency at ~400 Hz (Dunlap et al., 2012; Dunlap and Grant, 2014). In the rat, the otolith hair cell and calyceal synapses can generate responses above 100 Hz during mechanical probing of hair bundles (Songer and Eatock, 2013). Similarly, canal afferents of the turtle encode mechanical indentation stimuli of the posterior canal duct up to 100 Hz, where the gain of the afferent response increases with frequency across the tested bandwidth (Rowe and Neiman, 2012). During rotational stimuli, the gain of canal afferents relative to input rotational velocity in monkeys also increases with frequency and phase leads the stimulus over the entire reported bandwidth of 0–20 Hz (Sadeghi et al., 2007; Massot et al., 2011). These increasing gains (measured up to 20 Hz) indicate that the vestibular system can encode kinematic head stimuli above the tested bandwidth and thus above the frequencies that occur during normal movements and tasks.

Vestibular afferents may be tuned to specific frequencies of head movement. For example, mammalian canal afferents are categorized as regular or irregular based on their resting discharge variability (Goldberg, 2000). Regular afferents transmit more information at lower frequencies (<15 Hz) (Sadeghi et al., 2007), which is consistent with regular afferents being the primary contributors to the vestibulo-ocular reflex at frequencies <4 Hz (Minor and Goldberg, 1991; Chen-Huang et al., 1997). In contrast, irregular afferents exhibit a steeper increase in gain with frequency and a more pronounced phase advance than regular afferents (Sadeghi et al., 2007). Thus, irregular afferents are proposed to process high frequency information, which may be especially important when muscles need to respond to high frequency transient perturbations such as direct head impacts (Carriot et al., 2014).

Signals from both afferent types are further processed by the vestibular nuclei (VN). This processing depends on the afferent’s intrinsic membrane electrophysiology and can differ within and across nuclei (see review Straka et al., 2005). In the guinea pig medial vestibular nuclei (MVN), neurons are divided into two subtypes (A and B) that vary in spike shape, sensitivity to input currents and dynamic range (Ris et al., 2001; Beraneck et al., 2003). Type B neurons promote high frequency responses whereas type A neurons act as low-pass filters and are better suited to transmit the resting tonic activity of vestibular afferents (Ris et al., 2001). In contrast to MVN neurons, neurons in the lateral vestibular nucleus (LVN) have a lower sensitivity to input currents. LVN neurons appear to lack the decreasing-gain-with-increasing-frequency pattern observed in MVN neurons, and instead synchronize their firing to the input stimuli at a particular “cutoff” frequency (Uno et al., 2003). In monkey VN neurons, the increasing gain and phase described earlier mimic irregular afferents (Dickman and Angelaki, 2004; Massot et al., 2011). However, VN neurons transmit less information about head motion as compared individual canal afferents (Massot et al., 2011). Thus, it appears the type of processing vestibular afferent signals undergo depends on the nuclei and the specific neurons through which they travel.

Variations in the neural pathways may also contribute to the muscle specific characteristics of vestibular reflexes. Vestibulocollic pathways, which innervate the neck muscles, are mostly comprised of three-neuron-arcs that originate primarily from the MVN, descend via the bilateral medial vestibulospinal tracts, and have short (~8–10 ms) response latencies (Watson and Colebatch, 1998; Forbes et al., 2014). There are also indirect polysynaptic pathways mediating some vestibulocollic signals (reviewed in Wilson and Schor, 1999; Goldberg and Cullen, 2011). In comparison, vestibulospinal pathways, which innervate upper and lower limb muscles, originate from the LVN, and travel primarily ipsilaterally via the lateral vestibulospinal tract. Direct connections to limb motoneurons are exclusively excitatory while indirect connections via spinal interneurons can be both excitatory and inhibitory (Lund and Pompeiano, 1968; Wilson and Yoshida, 1969; Grillner et al., 1970; Shinoda et al., 1986; Davies and Edgley, 1994). In humans, lower limb muscle responses evoked by a vestibular stimulus exhibit longer latencies (~50–60 ms) than expected from a direct vestibulospinal connection (Britton et al., 1993; Fitzpatrick et al., 1994; Day et al., 1997; Ali et al., 2003; Lee Son et al., 2008), and this delay argues for additional processing of the evoked vestibular signals by central structures. Indeed, there is evidence that vestibular signals converge onto spinal interneurons, indicating that further processing of vestibular information may occur through local spinal pathways (Iles and Pisini, 1992; Thomas and Bent, 2013). Based on the data presented in this section, it appears that the vestibular organs are capable of sensing a wide range of input frequencies, but that the pathways then modulate and filter this response to suit the frequency required for the muscles to control their mechanical system (Forbes et al., 2013a).

## Extracting the frequency response of vestibulo-muscular systems

The frequency response of vestibular reflexes in muscles can be examined using mechanical perturbation and electrical stimulation techniques (see Figure 1). A common mechanical perturbation technique used to study VCR and vestibulo-ocular reflexes (VOR) is whole-body motion. Subjects are typically exposed to continuous sinusoidal or stochastic perturbations (e.g., rotation, tilt or translation) to characterize the transfer function, for instance, between head velocity and eye velocity when studying the VOR (Raphan et al., 1979; Robinson, 1981). Transient velocity steps can also be used to characterize the decay of vestibular reflex responses during otherwise constant velocity movements (Raphan and Cohen, 1985). Whole-body perturbations for the study of vestibular reflexes in axial or appendicular muscles may arguably be less relevant since there is no need to keep the head or body upright. They have nevertheless been used to study the frequency response of the VCR in decerebrate and alert animal preparations (Berthoz and Anderson, 1971; Ezure and Sasaki, 1978; Bilotto et al., 1982; Baker et al., 1985; Dutia and Hunter, 1985; Goldberg and Peterson, 1986; Keshner et al., 1992). These particular studies isolate the descending reflex pathways and provide important insight into the open-loop characteristics of neck vestibular reflexes. Whole-body linear accelerations have also been used in humans to induce vestibular responses in quiescent neck muscles, extra-ocular muscles, and upper and lower limb muscles (Greenwood and Hopkins, 1976; Aoki et al., 2001).

Isolated mechanical perturbations applied to the head, body or feet are perhaps more natural stimuli than whole-body perturbations for probing vestibular reflexes. Because these perturbations also stimulate somatosensory receptors, it can be difficult to isolate the vestibular contribution to postural control. During standing perturbations, afferent signals generated by ankle motion can be minimized by controlling the support surface tilt to match body sway (Nashner and Berthoz, 1978; Nashner et al., 1982). A comparison of these sway-referenced perturbations to natural perturbations, i.e., no sway referencing, allow the relative balance-related contributions of the somatosensory and vestibular systems to be estimated during standing balance and torso control (Fitzpatrick et al., 1996; Peterka, 2002; Goodworth and Peterka, 2009). An alternate approach relies on robotic balance systems to simulate normal stance where body motion is controlled by changes in isometric ankle torque (Luu et al., 2011). Robotic systems such as these not only emphasize vestibular contributions to balance, they allow the system’s mechanical properties (i.e., stiffness, damping and inertia) as well as the relationship between motor commands and sensory feedback to be manipulated and thus different aspects of postural control to be explored. While similar robotic techniques have yet to be developed for head, neck or torso postural control, the isolation of somatosensory contributions to gaze shifts has been implemented by counter rotating the body during head movements (Roy and Cullen, 2004).

Electrical stimulation of the vestibular organ is a non-invasive experimental technique used to probe human vestibular function (Fitzpatrick and Day, 2004). The applied current, which is delivered percutaneously using electrodes placed behind the ears, modulates the firing rates of vestibular afferents (Goldberg et al., 1984) and provides an artificial, isolated craniocentric vestibular error signal. The behavioral responses to electrical vestibular stimulation have been modelled (Fitzpatrick and Day, 2004) based on the distribution of vestibular afferents within the labyrinth and an assumption that all afferents (otoliths and semi-circular canals) are affected by the stimulus (Goldberg et al., 1984; Kim and Curthoys, 2004). The virtual head movement generated by binaural bipolar electrical stimulation (one of several possible electrode configurations) generates a perceived rotation about an axis directed posteriorly and superiorly by 18° relative to the Reid’s plane and a small lateral linear acceleration (Fitzpatrick and Day, 2004). In perception studies, this virtual rotation correlates maximally with real rotations when their two axes are co-linear (Day and Fitzpatrick, 2005), i.e., when the head is extended by 18°.

The vestibular error signals evoked by electrical stimulation have a strong effect on motor systems. Vestibular reflexes are evoked in ocular, axial and appendicular muscles, and manifest as changes in gaze and postural control in both humans and animals (Nashner and Wolfson, 1974; Lund and Broberg, 1983; Britton et al., 1993; Fitzpatrick et al., 1996; Watson and Colebatch, 1998; Watson et al., 1998; Ali et al., 2003; MacDougall et al., 2003; Aw et al., 2006; Ehtemam et al., 2012; Hsu et al., 2012; Zelenin et al., 2012; Kim, 2013). When the electrical stimulation is applied as sinusoidal or stochastic signals, the frequency response of vestibular reflexes can be characterized in a manner similar to that used with mechanical stimuli in many VOR and VCR studies (Fitzpatrick et al., 1996; Pavlik et al., 1999; Dakin et al., 2007, 2011; Forbes et al., 2013a). Unlike mechanical stimulation, electrical stimulation is not limited by the bandwidth of the mechanical system applying the perturbation or the neuromuscular system being investigated; thus, the frequency response of vestibular reflexes can be characterized over a larger bandwidth. Since concomitant activation of somatosensory afferents is limited to skin afferents behind the ears (which can be minimized with the application of a local anesthetic), electrical vestibular stimulation represents a powerful tool to examine the frequency response of vestibulomuscular systems across varying postural task conditions.

Despite these advantages, our understanding of electrical vestibular stimulation remains incomplete. For instance, it remains unclear whether otolith and semicircular canal afferents are stimulated equally. Some authors have suggested that only otolith responses are induced in humans (Cohen et al., 2012), whereas others have argued that semicircular canals are also involved (Curthoys and Macdougall, 2012; Reynolds and Osler, 2012). It is not our objective here to enter this debate; instead, we propose to rely on the consistent nature of the electrical vestibular stimulation to compare the frequency response characteristics of vestibular reflexes across postural conditions (e.g., different combinations of sensory feedback or the necessity to maintain balance) and muscle groups (e.g., appendicular and axial). Therefore, a substantial component of this review will consider observations made using electrical vestibular stimulation.

## Appendicular muscles: modular control at low frequencies

The primary function of vestibular reflexes in appendicular muscles is to generate muscle activity that maintains upright body posture and that ultimately contributes to stabilizing the head in space. Because the vestibular organs are fixed to the head, vestibular information must be transformed from the head reference frame before being used to generate appendicular muscle responses. For instance, postural sway evoked by electrical vestibular stimulation, which occurs primarily in the frontal plane when facing forwards, rotates with the orientation of the head relative to the feet (Lund and Broberg, 1983; Iles and Pisini, 1992; Britton et al., 1993). Consequently, the vestibular-evoked reflex responses in lower limb muscles are reversed between head-left and head-right postures (Britton et al., 1993; Dakin et al., 2007). This craniocentric response remains intact no matter how the head orientation is achieved, whether it be head only, trunk only or a combination of both (Lund and Broberg, 1983). Although these and other studies demonstrate the coordinate transformation that vestibular information undergoes and highlights its importance to standing balance of the whole body, other evidence suggests that this craniocentric principle may be less rigid than initially thought. When stance width is increased and the body becomes more stable in the frontal plane, the response magnitude to electrical vestibular stimulation becomes biased towards the sagittal plane such that changing head orientation results in a nonlinear relationship between head orientation and response direction (Mian and Day, 2014). These more recent results indicate that the balance system also integrates vestibular inputs with respect to whole-body stability (Mian and Day, 2014).

**Figure 2:**
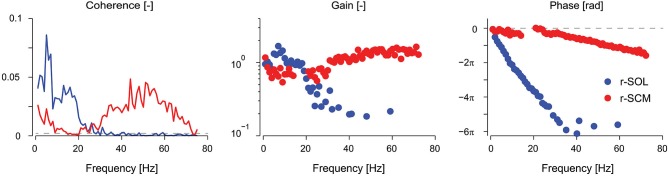
**Coherence, gain, and phase frequency estimates of vestibular reflexes for r-SOL and r-SCM muscles elicited by a 0–75 Hz stochastic electrical vestibular stimulus**. Vestibular reflexes in the r-SCM span a much wider bandwidth (~0–70 Hz) together with high gains and moderate phase lags relative to the r-SOL. The horizontal, segmented line in the coherence plot represents the level above which the coherence is significant. The horizontal, segmented line in the phase plot represents a phase of zero. r-SCM, right sternocleidomastoid; r-SOL, right soleus. (Data are adapted from Forbes et al., 2013a).

Vestibular reflexes in appendicular muscles contribute to balance over a bandwidth that, much like the vestibular system itself, extends beyond the assumed physiological range of vestibular signals (Armand and Minor, 2001; Huterer and Cullen, 2002). Vestibular reflexes evoked using stochastic electrical stimulation exhibit frequency components up to 25 Hz in lower limb muscles (see Figure 2; Dakin et al., 2007, 2010). The gain and phase of the reflexes resemble a low-pass filter with a cut-off of about 15 Hz and a phase inflection point at about 10 Hz (Dakin et al., 2007; Forbes et al., 2013a). The time domain estimate (i.e., cross-correlation) of the evoked muscle responses are equivalent to those from transient step-like stimulation: a biphasic response comprised of short and medium latency components (see Figure 1; Dakin et al., 2007). Frequencies above and below the 10 Hz inflection contribute primarily to the short and medium latency components respectively; however, the total response of the reflex is the linear sum of all frequencies and each frequency contributes to specific attributes of each component (Dakin et al., 2011).

This bandwidth of vestibular input to the appendicular muscles does not completely transfer to the mechanical response of standing balance. Vestibular input undergoes mechanical low-pass filtering when converted from lower-limb muscle activity to forces/moments and again from forces/moments to body sway, and results in forces/moments and body sway that are limited to <5 Hz and <2 Hz respectively (Fitzpatrick et al., 1996; Dakin et al., 2010). From a biomechanical control perspective, the high bandwidth of the vestibular input to the muscles is consistent with the electromechanical design principle that the dynamic range of a sensor (e.g., vestibular organ) must be greater than the dynamic range of the actuator (e.g., muscles), which in turn must be greater than the dynamic range of the underlying mechanical system (e.g., the body) to ensure effective and stable control (Franklin et al., 2009; Forbes et al., 2013a).

Vestibular reflexes in appendicular muscles also appear to be modulated by both additional sensory feedback and the postural task. For example, response amplitudes to electrical vestibular stimulation increase with altered ankle somatosensory cues and without vision (Nashner and Wolfson, 1974; Britton et al., 1993; Welgampola and Colebatch, 2001), whereas response amplitudes decrease with increasing stance width (Day et al., 1997) and the presence of external support (Britton et al., 1993; Fitzpatrick et al., 1994). Some of these effects however, are not always seen across the observed frequency bandwidth of vestibular reflexes. Fitzpatrick et al. (1996) found that the response gain of electrical vestibular stimulation in leg muscles did not change between 0 and 5 Hz with the eyes open or closed provided subjects stood on a rigid surface. In contrast, response gains increased at all frequencies when subjects stood on a compliant surface and then closed their eyes (Fitzpatrick et al., 1996). In line with this second observation, vestibular reflex gains in leg muscles increase at most frequencies from 0 to 5 Hz when subjects are instructed to minimize sway during electrical vestibular stimulation (Reynolds, 2010). This gain modulation also appears to extend above 5 Hz, where elevation-induced postural threats increase the gain and bandwidth of measured ground reaction forces evoked by electrical vestibular stimulation (Horslen et al., 2014).

**Figure 3:**
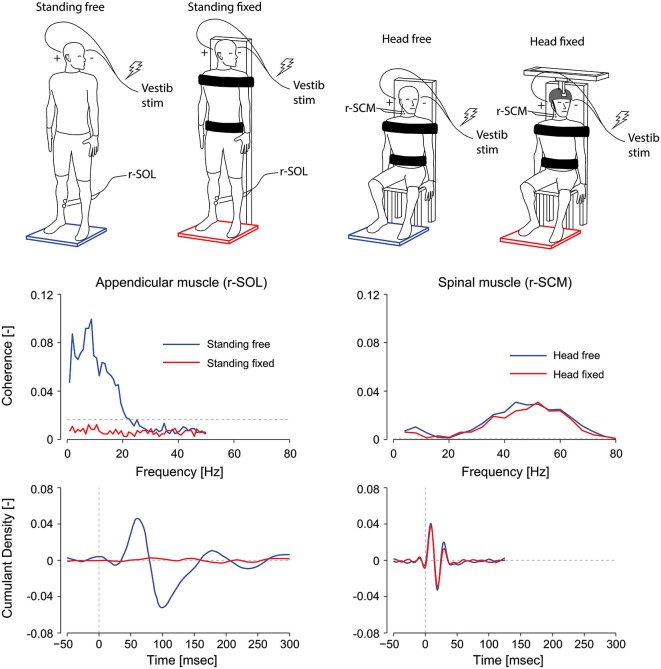
**Effect of postural task on appendicular and axial muscles**. In appendicular muscles (left plots), vestibular reflex frequency- and time-domain estimates (i.e., coherence and cumulant density respectively) are suppressed when a subject is standing with the torso fixed to a rigid support. In axial muscles (right plots), vestibular reflex responses are maintained when the subject’s head is fixed with respect to the torso. Thus, vestibular-evoked responses are present in axial muscles, unlike appendicular muscles, regardless of the postural task. r-SOL, right soleus; r-SCM, right sternocleidomastoid. The horizontal, segmented lines in the coherence plots represent the level above which the coherence is significant. (Data are adapted from Luu et al., 2012; Forbes et al., 2014).

Vestibular reflexes in appendicular muscles also vary based on their contribution to balance. For instance, lower limb responses in humans are entirely absent when subjects are seated, and are suppressed when standing subjects contract their leg muscles while being otherwise fully supported by a fixed backboard (see Figure 3; Britton et al., 1993; Fitzpatrick et al., 1994; Luu et al., 2012). More notably, vestibular reflexes are also suppressed when subjects balance a body-equivalent inverted pendulum while being externally supported, a task where somatosensory information mimics normal standing but vestibular information is incongruent (Fitzpatrick et al., 1994). The latter observations suggest that in addition to the need for a muscle to contribute to balance, a muscle must remain relevant to balance control for vestibular reflexes to be evoked. More recent work has also shown that this suppression depends on the congruency of the motor and sensory signals (Luu et al., 2012). When standing subjects balanced a robotic platform to which they were rigidly strapped, vestibular input decreased in lower limb muscles in most subjects as the computer imperceptibly took over control of balancing the platform. Based on these results it appears that vestibular input to balance control varies with the congruency of the sensory feedback and the underlying motor behavior (Luu et al., 2012). This principle appears to apply to any appendicular muscle, since vestibular responses can be evoked provided the muscles are directly involved in the task to maintain balance; or in other words, that the force output of the muscle contributes to balancing the body in space (Britton et al., 1993; Luu et al., 2012).

The task dependence of vestibular reflexes in appendicular muscles extends beyond standing balance. During walking in humans, vestibular reflex responses are dynamically modulated in all locomotor muscles about the ankle, knee and hip joints (Iles et al., 2007; Blouin et al., 2011; Dakin et al., 2013). The reflex responses vary with the phase of the gait cycle and also vary between muscles: ankle muscle responses (e.g., soleus and medial gastrocnemius) are typically strongest at heel strike, whereas lateral hip muscle responses (e.g., gluteus medius) are active just before and after heel contact. In addition, the reflex responses do not vary strictly with muscle activation level, which suggests that phase- and muscle-specific responses during walking are organized according to a muscle’s functional role in whole-body stabilization (Blouin et al., 2011; Dakin et al., 2013).

The flexible nature of vestibular-evoked responses in appendicular muscles provides a convenient platform to address unanswered questions regarding the human balance system. For example, although it is clear that vestibular information must be congruent with the postural task to evoke vestibular reflexes, the source and relevance of sensory feedback required to engage the vestibular control of standing balance remains to be determined. Similarly, the potential influence of the mechanical properties of standing balance (i.e., stiffness, damping and inertia) on the vestibular—and more generally the sensorimotor—control of standing has yet to be established. For instance, increased muscle stiffness, as experienced by Parkinson patients, could be simulated in healthy controls in order to understand its effects on sensorimotor processing during standing balance. Robotic balance simulators (i.e., Luu et al., 2011) are viable platforms to explore these questions, and their continued development and application will generate insight into the central processing of vestibular-evoked responses.

## Axial muscles: robust high frequency spinal stabilization

Most studies of vestibular reflexes in axial muscles have focused on the cervical spine and the VCR. The primary function of the VCR is to stabilize the head in space by generating muscle contractions that oppose the instantaneous head motion. This was first demonstrated by electrically stimulating individual cat semicircular canals and generating stereotyped head movements opposite to those that would activate the canals (Suzuki and Cohen, 1964). Descending vestibular signals innervate neck motor neurons with muscle-specific patterns of inhibitory/excitatory connections (Shinoda et al., 1992, 2006; Perlmutter et al., 1998) that are thought to reflect the function of individual muscles in maintaining the head stable in space (Wilson and Schor, 1999). This is in agreement with the observation that neck muscles generate preferential VCR response vectors during whole-body rotation of the cat (Baker et al., 1985; Keshner et al., 1992).

Although these preferred VCR response vectors evoke highly stereotyped muscle-specific EMG patterns (Peterson et al., 2001), little is known about how these response patterns vary with head posture. In cat dorsal neck muscles, EMG activity varies linearly with changing head yaw orientation during whole-body rotations about the same axis (Banovetz et al., 1995). However, the head must be reoriented up to 25 degrees in order to shift the whole-body-rotation driven muscle activity to a degree greater than the standard error of the population. These results are similar to recent observations in humans showing that electrically evoked VCRs do not vary across head yaw reorientations of up to 60 degrees from neutral (Forbes et al., 2013a, 2014). Considering that the origin and/or insertion points of neck muscles move with the head and neck, it is possible that VCR responses remain close to a muscle’s preferred direction in the neutral posture of the head in order to generate a compensatory activity consistent with the craniocentric vestibular error signal.

Dynamical models of the VCR have been created and estimating the open-loop characteristics of these models has been the focus of many studies in animals exposed to whole-body movements (Berthoz and Anderson, 1971; Ezure and Sasaki, 1978; Bilotto et al., 1982; Baker et al., 1985; Dutia and Hunter, 1985). Across most frequencies, VCR responses can be explained by the direct trisynaptic pathways thought to mediate this reflex. At low frequencies (0.01–0.1 Hz), however, muscle responses lag behind input acceleration by up to 150° and lag behind vestibular nucleus neuron responses by up to 90° (Shinoda and Yoshida, 1974; Ezure and Sasaki, 1978). To explain this phenomenon, Ezure and Sasaki (1978) proposed neural integration of the descending vestibular signals, and the existence of indirect polysynaptic neural circuits to accomplish this integration is supported by the absence of response variations during medial longitudinal fasciculus transection in the cat (Miller et al., 1982; Thomson et al., 1995). The exact structures involved in these indirect pathways, however, remain uncertain (see Wilson and Schor, 1999; Goldberg and Cullen, 2011). In humans, the possibility of multiple pathways underlying the VCR is supported by an abrupt gain and phase shift in the frequency response of the sternocleidomastoid and splenius capitis muscles during electrical vestibular stimulation (see Figure 1; Forbes et al., 2013a). Although these abrupt changes could be due to destructive interference of two reflex pathways, similar to those observed in mechanically evoked stretch reflexes of the human wrist (Matthews, 1993), additional work is needed to confirm this hypothesis.

In humans, the electrically evoked VCR is a short-latency (~10 ms) short-duration biphasic waveform (see Rosengren et al., 2010 for review). In the sternocleidomastoid muscle, the peaks of this biphasic waveform occur at about 10–13 ms and 21–23 ms (Rosengren et al., 2010; Forbes et al., 2014) and the frequency content of the response extends up to 70 Hz (see Figure 2; Forbes et al., 2013a). As noted for appendicular muscles, this wide bandwidth is thought to facilitate control over the high frequency dynamics (up to 20 Hz) of head-neck stabilization (Viviani and Berthoz, 1975; Grossman et al., 1988; Pozzo et al., 1990). The gain and phase of the VCR varies between muscles, being primarily high frequency in the sternocleidomastoid muscle (30–70 Hz) and primarily low frequency (0–20 Hz) in the splenius capitis muscle (Forbes et al., 2013a, 2014). Regardless of its frequency response, the amplitude of the electrically evoked VCR scales with the amplitude of the background neck muscle activity (Watson and Colebatch, 1998) and is absent when the muscle is not active (Watson and Colebatch, 1998; Forbes et al., 2014).

There is substantial evidence that VCRs are modulated by the concurrent stabilization task, although this modulation appears to be limited primarily to low frequencies. During stabilization of the head-neck during trunk perturbations, the VCRs are thought to dampen oscillations of the otherwise under-damped mechanics of the passive head-neck system (Keshner et al., 1995; Peng et al., 1996). This damping is thought to occur primarily between 1–2 Hz in alert animals and humans (Baker et al., 1985; Goldberg and Peterson, 1986; Keshner et al., 1995; Forbes et al., 2013b). Visual fixation improves head-in-space stabilization in humans (Guitton et al., 1986; Goldberg and Cullen, 2011) and may be driven by increased VCR contributions (Forbes et al., 2013b). During anterior-posterior perturbations, there is a shift from minimization of head-in-space motion to head-on-torso motion as the perturbation exceeds the system’s natural frequency (~2–3 Hz) (Forbes et al., 2013b). At perturbation frequencies above 2–3 Hz, long phase lags caused by reflex time delays would cause the VCR to destabilize the system, and as a result the CNS attenuates (but does not inhibit) these neural contributions (Kearney et al., 1997; van der Helm et al., 2002; Schouten et al., 2008).

In contrast to the VCR’s low-frequency response dynamics, the VCR’s high-frequency (i.e., short latency) response dynamics in the sternocleidomastoid muscle are insensitive to changes in vision, external support, stance width and posture (Watson and Colebatch, 1998; Welgampola and Colebatch, 2001). These insensitivities led us to question whether the requirement to maintain an upright or elevated head posture—a task that relies on vestibular information—governs the high frequency contribution of the VCR response to muscle activity. To answer this question, we fixed the head and torso of subjects and asked them to generate isometric neck muscle contractions. Although subjects activated their neck muscles, this activity was irrelevant to the maintenance of an upright head posture (see Figure 3). This condition is analogous to subjects being seated and contracting lower limb muscles when evaluating vestibular task-dependency in appendicular muscles. Unlike the attenuated vestibular-evoked responses observed in appendicular muscles, the VCR responses remained present even with the head fixed (Forbes et al., 2014). Considering that the VCR forms a closed-loop system wherein its output, i.e., neck muscle driven motion, directly affects the vestibular input, a robust VCR makes sense and ensures a highly effective response to external disturbances. A significant reduction in the VCR, however, was observed in the splenius capitis muscle during head fixation (Forbes et al., 2014). This reduction of the splenius VCR response was detected only for the lower frequency response of the VCR (below 20 Hz), which is very weak for the sternocleidomastoid muscle (see Figure 2). We propose the effects of task dependency (i.e., muscle activity being relevant to vestibular afference) reported in the appendicular muscles and splenius capitis are expressed primarily in the low frequencies of the vestibular reflex response.

The amplitude of the VCR response (at both low and high frequencies) also varied little between isometric tasks involving different neck muscle activation patterns with equivalent activation levels. For example, similar VCR responses were observed in the sternocleidomastoid muscle during isometric contractions in flexion and yaw, as well as in the splenius capitis muscle during isometric contractions in extension and yaw. This low sensitivity to the combination of muscles being activated highlights the flexibility in neck muscle control, where the activation of a group of muscles does not have strong reciprocal (inhibitory or excitatory) connections to other muscles (Forbes et al., 2014). One exception to this task insensitivity was observed in the sternocleidomastoid during neck muscle co-contraction. Attenuated VCR responses during co-contraction are nevertheless consistent with the goal of head-on-torso stabilization: the increased neck stiffness caused by co-contraction presumably tightens head-to-torso coupling and an un-attenuated VCR response would oppose this coupling and could be detrimental to head-neck stabilization.

Our knowledge of vestibular reflexes to thoracolumbar muscles is limited in comparison to cervical muscles, although there is evidence that vestibular input plays a role in upper body control. In cats, vestibulospinal spinal neurons also form monosynaptic excitatory and inhibitory connections with thoracic spinal motoneurons (Wilson et al., 1970a,b) and responses appear to originate in particular from otolith input (Brophy et al., 1997). In humans, erector spinae muscle responses to electrical vestibular stimuli appear to be organized together with the lower limb muscle responses during standing balance (Ardic et al., 2000; Ali et al., 2003). The latencies associated with these responses are consistent with a progressively descending vestibular signal, occurring earlier in paraspinal muscles (~61 ms) than in lower limb muscles (~85 ms) (Ardic et al., 2000; Ali et al., 2003). However, the frequency response of vestibular reflexes in erector spinae muscles have a reduced bandwidth (0–15 Hz) and lower gain roll off (~3 Hz) compared to lower-limb muscles (bandwidth: 0–25 Hz, gain roll off: ~15 Hz) and neck muscles (bandwidth: 0–70 Hz, gain roll off: not observed) (Forbes et al., 2013a). The ability to respond effectively to only low frequency vestibular input suggests that thoracolumbar muscles may have a limited functional contribution to standing balance compared to the contribution of lower limb and neck muscles. If we postulate that lower limb muscles balance the trunk, and neck muscles fine-tune this balance for the head, then low frequency coupling of the thoracolumbar spine may be all that is needed to maintain a stiff enough trunk for the system to function.

The relative insensitivity of the VCR to postural task may provide an opportunity to examine several methodological and fundamental questions regarding vestibular sensorimotor processing in humans. For instance, the possibility of evoking the VCR in active neck muscles while the head is immobilized facilitates the experimental and clinical testing of these responses. It also permits investigating how vestibular inputs to the neck motoneurons interact with sensory or descending motor inputs to these motoneurons under well-controlled conditions. For example, potential modulations of the VCR during whole-body motion can be readily tested with electrical vestibular stimuli while keeping the head fixed with respect to the torso. Furthermore, the influence of neck somatosensory inputs on the electrically-evoked VCR could be assessed while volunteers maintain a constant level of neck muscle activity with the head fixed in space. Resolving these important issues will advance our understanding of the vestibular control of neck muscles and potentially lead to applications for patients suffering from head-neck sensorimotor disorders. Future development of head-neck robotic devices, similar to the standing balance robot developed by Luu et al. (2011), could prove similarly useful in exploring the control of neck posture and gaze.

## Conclusions

Prior studies of the vestibular system’s contribution to postural control have been limited to frequency bandwidths below 20 Hz. More recent work, however, suggests that vestibular contributions to postural muscles can be measured up to 25 Hz in appendicular muscles and 70 Hz in neck muscles. We argue that this system dependency (i.e., whole-body postural control vs. head postural control) is related to the bandwidth of the mechanical system under control, which for the head-neck system during voluntary movements and imposed force perturbations is up to 5 times wider compared to the whole body during standing balance (Viviani and Berthoz, 1975; Pozzo et al., 1990). It remains unclear how the central nervous system controls the required bandwidth, although neural filtering, created by variations in the dynamics of the VN or the spinal circuitry mediating the descending vestibular signals, may be involved. Further animal studies are needed to evaluate these and other possibilities.

Recent studies also show a frequency dependent modulation of vestibular signals in both appendicular and axial muscle responses at low frequencies (<25 Hz). Although modulation of higher frequencies (up to 70 Hz) in neck muscles was observed in some conditions (i.e., neck muscle co-contraction), this part of the neck vestibular reflexes frequency response were maintained even in conditions where the muscle did not directly contribute to postural control. The absence of a reduced high-frequency VCR response with the head fixed parallels the response of the electrically evoked VOR, which occurs regardless of the functional or postural state of the head and body (Aw et al., 2006). Similar to the VCR, the VOR in extraocular muscles is short-latency and short-duration (Weber et al., 2012), indicating that the VOR is also driven primarily by high frequencies (likely 30–70 Hz and possibly higher). It is noted however, that the frequency response of the VOR has yet to be characterized above 20 Hz.

Furthermore, the VOR is subject to suppression during gaze shifts and gaze pursuit, where an intact VOR would be counterproductive to the intended change in gaze or ongoing tracking (see review, Cullen and Roy, 2004). Whether a similar suppression of the VCR occurs, for example during self-generated head movements where reafferent vestibular information is substantially reduced, remains unknown (Goldberg and Cullen, 2011; Cullen, 2012). Indeed, it is suggested that an intact VCR during self-generated movements may continue to dampen the head-neck system (Goldberg and Cullen, 2011), since increased head oscillations are observed during voluntary movement in various animal preparations following canal plugging (Schor, 1974; Baker et al., 1982; Paige, 1983). Comparing the electrically evoked VCR during self-generated and passively-imposed movements should provide a clearer answer regarding potential suppression of the VCR during self-generated head movements. Regardless of the exact suppression mechanisms, given that both neck and oculomotor systems receive similar descending input from several neural structures (Freedman et al., 1996; Corneil et al., 2002; Elsley et al., 2007; Knight and Fuchs, 2007), and are activated in a coordinated manner to generate rapid gaze shifts (Guitton, 1992; Guitton et al., 2003), these two systems may work together to generate effective vestibulo-motor responses for gaze control.

The data reviewed here have important implications for understanding the role of the vestibular system in controlling posture and gaze. Indeed, studying separately individual effectors controlled by the vestibular system would lead to diverging conclusions regarding its role in a specific task. A similar statement could be made for the examined bandwidth of the vestibular reflexes. We instead propose an integrative approach that simultaneously examines the complete frequency response of vestibular reflexes in appendicular, spinal and extraocular muscles. Using combinations of robotic systems and electrical vestibular stimulation, it may be possible to evaluate the relative dependence or independence of vestibular input on axial and appendicular systems. The evaluation of vestibular input expressed in multiple motor systems will provide a more comprehensive understanding of how the vestibular system contributes to the complex behavioral tasks of daily living.

## Conflict of interest statement

G.P. Siegmund owns shares in a consulting company, and both he and the company may derive benefit from being associated with this work. All other authors declare that the research was conducted in the absence of any commercial or financial relationships that could be construed as a potential conflict of interest.
